# Genome Sequence of *Pantoea ananatis* Strain AMG 501, a Plant Growth-Promoting Bacterium Isolated from Rice Leaves Grown in Paddies of Southern Spain

**DOI:** 10.1128/genomeA.00848-17

**Published:** 2017-08-24

**Authors:** Esaú Megías, Fábio Bueno Reis Junior, Renan Augusto Ribeiro, Francisco Javier Ollero, Manuel Megías, Mariangela Hungria

**Affiliations:** a Facultad de Biología, Departamento de Microbiología, Universidad de Sevilla, Seville, Spain; b Embrapa Cerrados, Soil Microbiology, Planaltina, Federal District, Brazil; c CNPq, SHIS QI 1 Conjunto B, Lago Sul, Brasília, Federal District, Brazil; d Embrapa Soja, Soil Biotechnology, Londrina, Paraná, Brazil

## Abstract

*Pantoea ananatis* AMG 501 is a plant growth-promoting bacterium isolated from rice leaves. Its genome was estimated at 5,102,640 bp with 4,994 coding sequences, encompassing genes related to the metabolism of carbohydrates, to the synthesis of auxins, siderophores, and homoserine lactones, and to the type I, II, III, IV, and VI secretion systems.

## GENOME ANNOUNCEMENT

We have extensively isolated rhizospheric and endophytic *Pantoea* spp. from rice (*Oryza sativa* L.) paddies of the Guadalquivir River marshes in southern Spain. Previously, we sequenced the genomes of the root endophyte *Pantoea ananatis* strain AMG521 (1) and of the rhizospheric *Pantoea* sp. strain 1.19 (2), and now we present the genome of *P. ananatis* strain AMG 501, an endophyte of rice leaves. Plant growth-promoting properties of AMG 501 *in vitro* include the production of siderophores, auxins (indole acetic acid-IAA, 67.76 mg mL^−1^), and cellulase. The bacterium synthesizes at least 14 different molecules of *N*-acyl-homoserine-lactones (HSLs). The rhizospheric and foliar application of AMG 501 increases biomass and/or grain production—by 10 to 60%—of legumes (alfalfa, *Medicago sativa*), pastures (*Urochloa brizantha*), vegetables (tomato, *Solanum lycopersicum*), and cereals (rice).

Total DNA was extracted using the DNeasy blood and tissue kit (Qiagen) and processed on the MiSeq platform (Illumina) at Embrapa Soja, Londrina, Brazil. Paired-end reads obtained by shotgun sequencing allowed a genome coverage of 65-fold. The FASTQ files were *de novo* assembled with the A5-miseq pipeline (3). The genome was estimated at 5,102,640 bp, assembled in 51 contigs, with a G+C content of 53.6 mol%. *P. ananatis* AMG 501 carries two plasmids of about 100 and 150 Mb each. The average nucleotide identity values of AMG 501 compared with the whole genomes of *P. ananatis* LMG2665^T^ and *P. ananatis* AMG521 were 99.09 and 96.42%, respectively.

Sequences were submitted to the RAST server (4), and the annotation identified 4,994 DNA coding sequences, with 58% classified in 535 subsystems. The major categories were of carbohydrates (12.5%), amino acids and derivatives (8.9%), and proteins (4.9%). Several genes related to the metabolism of monosaccharides, disaccharides, and polysaccharides were detected in AMG 501 and were more abundant than in *P. vagans* and *P. agglomerans* (5).

AMG 501 carries genes belonging to secretion systems of types I, II, III, and IV. In addition, similar to the genomes of *P. ananatis* strains (6), there are genes of the type VI secretion system (TSS6) that usually function as injectisomes for effector proteins. In AMG 501, TSS6 includes the IncF conjugal transfer system (Tra operon, 18 genes) and the IncI plasmid conjugative transfer system (7).

There are at least 18 genes of the biosynthesis of dihydroxamate siderophores and ferrichrome (FhuC and FhuB proteins and aerobactin) (8, 9). Compatible with the numerous HSLs identified *in vitro*, AMG 501 carries the autoinducer system 2 (AI-2) (*lsrACDBFGE* operon), a quorum-sensing signaling molecule proposed to be involved in interspecies communication (10).

Genes of the IAA metabolism, tryptophan synthase (alpha and beta chains), and *ysnE* (IAA-acyltransferase), similar to those found in *Bacillus amyloliquefaciens* (11), indicate an alternative route in the biosynthesis of auxins. Noteworthy also are the genes related to stress response (3.3% of the genome), including osmotic stress, oxidative stress, cold and heat shock proteins, choline, betaine, glutathione, beta-glucans, and trehalose synthesis, in addition to aquaporin Z.

### Accession number(s).

This whole-genome shotgun project has been deposited at DDBJ/EMBL/GenBank under accession no. NIRH00000000, SUBID no. SUB2752749, BioProject no. PRJNA389525, and BioSample no. SAMN07201750. The version described in this paper is the first version, NIRH01000000.
